# Country-Wide qPCR Based Assessment of *Plasmodiophora brassicae* Spread in Agricultural Soils and Recommendations for the Cultivation of Brassicaceae Crops in Poland

**DOI:** 10.3390/pathogens9121070

**Published:** 2020-12-20

**Authors:** Anna Czubatka-Bieńkowska, Joanna Kaczmarek, Katarzyna Marzec-Schmidt, Anna Nieróbca, Agnieszka Czajka, Małgorzata Jędryczka

**Affiliations:** 1 Department of Vegetable and Ornamental Plants Protection, Research Institute of Horticulture, Kościuszki 2, 96-100 Skierniewice, Poland; anna.czubatka@inhort.pl (A.C.-B.); agnieszka.czajka@inhort.pl (A.C.); 2 Institute of Plant Genetics, Polish Academy of Sciences, Strzeszynska 34, 60-479 Poznań, Poland; jkac@igr.poznan.pl; 3 Department of Soil and Environment, Swedish University of Agricultural Sciences, Gråbrödragatan19, 532 31 Skara, Sweden; Katarzyna.Marzec-Schmidt@slu.se; 4 Department of Agriculture, Pope John Paul II State School of Higher Education, ul. Sidorska 95/97, 21-500 Biała Podlaska, Poland; szewc@iung.pulawy.pl; 5 Institute of Soil Science and Plant Cultivation, State Research Institute, Czartoryskich 8, 24‑100 Puławy, Poland

**Keywords:** clubroot, pedology, soil infestation, soil mapping

## Abstract

Clubroot is a damaging disease of oilseed rape and vegetable brassicas worldwide, caused by the soil-borne protist *Plasmodiophora brassicae* Wor. Due to the long life of resting spores, the assessment of the pathogen abundance in agricultural fields can serve as a guideline for disease control at the country-wide level or the regional scale. Between 2013 and 2019, we collected 431 soil samples from fields cultivated with Brassicaceae crops throughout 16 provinces of Poland. The samples were subjected to qPCR based analysis of *P. brassicae* DNA concentration. From these data, the spore loads and gene copies g^−1^ soil were calculated and used to produce an assessment of the current clubroot risk potential at a country-wide and regional scale. The country-wide map, showing the spread of the pathogen in agricultural soils, was made using ArcGis software package implementing the interpolation with the Inverse Distance Weight method. The calculation of gene copies specific to *P. brassicae* helped to formulate the recommendations for farmers in respect to the cultivation guidelines. It showed a high risk of yield losses in defined regions of north, south-west and central Poland and an urgent need to undertake intensive preventative measures.

## 1. Introduction

The genus *Brassica*, especially cabbage and oilseed rape, represents a significant segment of agricultural production in many countries, including Poland. In recent years, over 20% of the vegetables grown in Poland are brassicas and almost 13% of its agricultural land is occupied by oilseed rape, predominantly the winter form. In 2019, there were about 20 thousand ha of cabbage, 9.4 thousand ha of cauliflower and almost 0.9 million ha of oilseed rape, with the highest intensity of their production in Lower Silesia and the lowest in Swietokrzyskie Province. Since 2005, this significant acreage has ranked Poland in 4th place in the European Union for the production of oilseed rape and 7th place globally. However, the intensification of production of these crops brings serious phytopathological problems that can affect plant yield and quality.

Clubroot caused by a protist *Plasmodiophora brassicae* is a damaging soil-borne disease of *Brassica* crops, first described in 1878 by Woronin [1]. It is widespread throughout the world; it has been found in 35 European countries, 11 Asian countries, 10 countries in North and South America, three regions of Oceania, as well as in Africa [2]. Clubroot is considered a pandemic disease causing severe yield losses of *Brassica* crops—about 15% worldwide. Very high crop losses were reported in all countries growing vegetable brassicas and oilseed rape, primarily in Canada [3] and European Union [2]. In fields heavily infested by *P. brassicae*, there were reports of plant yield loss ranging from 30% up to total crop loss [4,5,6]. Thus, clubroot is considered one of the most economically important threats to Brassicaceae production [7,8].

Clubroot is one of the major challenges for brassicas cultivated in Poland. The disease is distributed over a large area of cabbage [9] and oilseed rape cultivation [10]. The severity of its occurrence has been very diverse in different regions of the country [11,12,13]. Unlike some other pathogens of Brassicaceae crops [14], the sexual stage of *P. brassicae* has not yet been firmly established. Despite this, the pathogen shows great genetic variability and a great number of different pathotypes have been found worldwide, and within Poland [9,15,16,17]. In 2012, in Poland the pathogen appeared on more than 250 thousand ha of soil cultivated with oilseed rape and this territory is still expanding, making clubroot one of the most important limiting factors for the production of *Brassica* crops in many regions of the country [18]. The primary reason for this phenomenon is the dependence on monocultures and lack of adherence to the principles of crop rotation.

*Plasmodiophora brassicae* is an endemic organism occurring in peat soils, both in low and high bogs exploited for the production of seedlings of *Brassica* vegetables [19]. The pathogen infects the roots of host plants; primary infection can occur in many species, but secondary infections are restricted predominantly to the Brassicaceae family [20,21]. Under the influence of root exudates from a host plant, resting spores distributed in the soil start releasing zoospores, which penetrate the root hairs and rhizodermal cells and fill them with plasmodia [22]. Host plant development is reprogrammed by the pathogen which diverts plant nutrition to infection sites [23,24]. With the development of the pathogen inside plant tissues, the ability to uptake nutrients and water by infected roots is greatly reduced. Moreover, the size and number of infected cells increases (hypertrophy and hyperplasia) forming galls and clubs on the whole root system. Infected plants rapidly wither, stunt, ripen prematurely and even die, if the infection is severe [25]. Conditions that foster the development of the disease are high soil moisture, acidic pH, and a soil temperature between 23–26 °C [9,26,27]. Zoospores are mobile in water, so the pathogen has a great ability to spread over large areas via drainage systems [28]. Furthermore, the pathogen easily and quickly spreads with soil particles contaminating agricultural machinery, wheels, and even the shoes of the farmers [29]. Other methods of pathogen spread have been examined, including its dispersal in dust occurring as an external contaminant of seeds and tubers [30] as well as in manure from livestock fed on *P. brassicae*-infested feed [31]. Moreover, resting spores of *P. brassicae* can survive and remain viable in the soil for 20 years without the presence of host plants [20]. Other causes increasing the severity of the disease in recent years include global warming, the lack of effective chemicals to reduce the pathogen, and the pathogen overcoming the resistance present in some cultivars [32]. 

Due to the longevity of resting spores and their high resistance to chemical treatments, a diagnosis of the level of *P. brassicae* infestation in soil before the decision to cultivate *Brassica* crops is one of the most effective ways to avoid yield losses [33]. Early detection of the pathogen enables the cultivation of other crops and counteracts the further spread of the disease [13]. Serious difficulties with high prevalence and severity of *P. brassicae* on Brassicaceae crops, especially on cultivated brassicas, requires quick and sensitive methods of pathogen detection. The visual recognition of clubroot galls comes too late for farmers to undertake the appropriate measures to control the disease. In this case, chemical treatment can be used but its application is technically and economically challenging. Moreover, the final effectiveness may be insufficient and can cause a potential threat to the environment [2,34]. Infection by the pathogen is enhanced by soil characteristics such as acidic pH, poor structure, increased temperature and impeded drainage [35]. These conditions vary within many arable fields. 

The oldest method to estimate the number of pathogen spores in the soil was described in 1933 by Fedotova [36]. Spores were counted in the aqueous soil solution using an optical microscope. This method was enhanced by improving the extraction and separation of spores from soil and the use of phase contrast or fluorescence microscopes [37,38]. An old but still widely used method to indirectly detect *P. brassicae* in the soil is a bioassay with different sets of susceptible plants. The evaluation of the disease incidence and severity of the symptoms on plant roots is completed after 5–6 weeks [39,40]. Such a bioassay detects spores at a concentration between 10^2^ and 10^5^ spores g^−1^ soil [41]. Even though these assays are effective and reliable methods, they are also space and time consuming, so faster and more accurate methods have been elaborated.

In 1989, Lange et al. [42] used dot immunoblotting to identify clubroot in plant roots. Six years later a method of spore detection in soils by immunoassay was elaborated [43]. However, immunoassays require monoclonal antibodies highly specific to *P. brassicae*. Although there is a report showing new insights in designing this antibody [44], serological assays have not been used as routine tests for the detection of *P. brassicae*.

Current assays are based on molecular biology techniques. Over the last twenty years, PCR-based methods have been applied to detect *P. brassicae* in many studies. These assays are still developed and used due to their speed, specificity, and sensitivity to the pathogen. The first molecular approach to detect *P. brassicae* in soil was based on nested-PCR, with primers specific to the DNA sequence of *P. brassicae* [45,46,47,48] and they were followed by other PCR-based tests, such as the one developed by Cao et al. [49]. All these methods were used for the detection of the pathogen from plant and soil samples. The methods by Faggian et al. [46] and Cao et al. [49] enabled the detection of 1 × 10^3^ *P. brassicae* spores g^−1^ soil. Staniaszek et al. [47] detected the pathogen in peat moss substrate and on artificially infested mineral field soil when the concentration of spores exceeded the abovementioned concentration. Wallenhammar and Arwidsson [48] measured the abundance of spores in different naturally infested soils and the results were presented in the relation to disease severity index (DSI). The pathogen was detected in soils where DSI was higher than 21, which corresponds to the results obtained by Staniaszek et al. [47]. However, since nested PCR detects the pathogen, without its quantification, this method cannot be used to track the progress of the control treatments or to evaluate the dynamics of spore multiplication. Moreover, the two-step PCR procedure increases the cost and it is time-consuming and prone to additional mistakes.

Since 2010, it has been possible not only to detect the presence of the pathogen but also to determine its quantity using the real-time PCR method using the analysis of fatty acids as a biomarker [50]. However, till now there is no report about the use of this method in naturally infested soils. Currently, the most sensitive method detects 500 resting spores in 1 g of naturally infested soil; this method is based on quantitative real-time PCR with TaqMan probes specific to *P. brassicae* ITS ribosomal DNA region [51]. In 2013, Li et al. [52] reported a cheaper method of real-time PCR detection and quantification with the use of SYBR Green, but the detection limit was two times higher than that achieved by Wallenhammar et al. [51]. The limit of detection of the Loop-mediated isothermal DNA amplification (LAMP) was ten times higher as compared to the use of TaqMan chemistry (5000 spores vs. 500 spores, respectively). On the other hand, the LAMP method is much cheaper, easier, and faster than the real-time PCR technique [53]. Currently, a number of methods are available and the choice of method depends on the aim of the study.

An increasingly popular focus of disease management in recent years is testing soil for the presence of *P. brassicae* spores in fields dedicated to the cultivation of brassicas. Such studies are routinely done in Sweden by accredited laboratories such as Eurofins (http://www.eurofins.se). Our study is the first attempt at a country-wide qPCR based assessment of *P. brassicae* spread in agricultural soils of Poland, enabling a comparison with the situation in the other countries or areas abroad. Based on these results the recommendation for the cultivation of oilseed rape and vegetable brassicas has been elaborated for all regions of Poland.

## 2. Results

### 2.1. Establishment of the Linear Regression between Microscope Observations and qPCR Results

**Figure 1:**
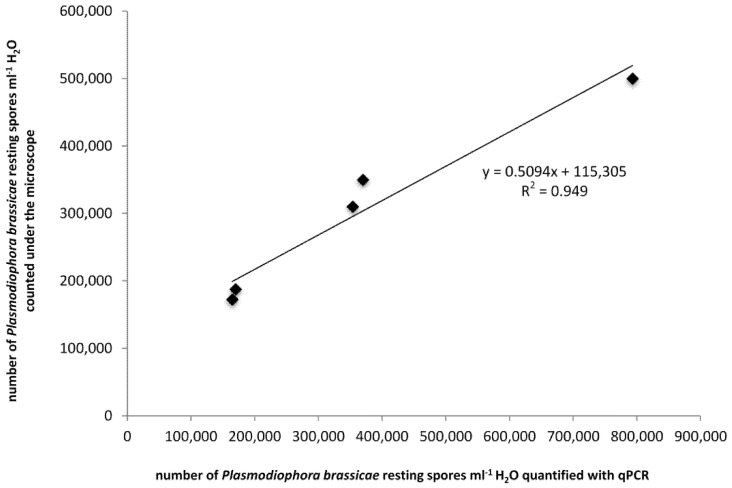
Linear regression between spore counts and real-time qPCR results using resting spores obtained from Chinese cabbage roots infected by five isolates of *Plasmodiophora brassicae* (◆) obtained from clubs on oilseed rape (*Brassica napus* L.) roots from different regions of Poland.

Spores extracted from Chinese cabbage roots infected with five isolates of *P. brassicae* obtained from clubs on roots of oilseed rape grown in different regions of Poland were quantified by counting under the microscope and a real-time qPCR method. The latter assay detected and quantified the DNA of all tested isolates. The correlation between these two methods was linear, and the correlation coefficient R^2^ was equal to 0.949 (Figure 1), which confirms the effectiveness of the qPCR method to precisely quantify the number of *P. brassicae* resting spores in tested samples.

### 2.2. Quantification of P. brassicae in Soil Samples Using Real-Time qPCR 

Over the years 2013 to 2019, 431 soil samples were collected from agricultural soils, mainly under oilseed rape and Brassicaceae vegetable cultivation, across all sixteen provinces in Poland. After DNA extraction, the amount of *P. brassicae* in each sample was quantified using real-time qPCR and expressed as fg of pathogen DNA g^−1^ soil. The quantification was experimentally confirmed and precise above the level of 5 fg g^−1^ soil, which is the equivalent of 1300 gene copies or 3000 spores g^−1^ soil [51]. Calculation using the Inverse Distance Weight Interpolation of the ArcGIS software suite (ESRI Polska Sp. z o.o., Warszawa, Poland) measured the approximate areas and shares of arable land in Poland attributed to different concentrations of *P. brassicae* DNA (Table S1). The highest percentage area (20.9%) was attributed to a pathogen DNA concentration 250–1000 fg g^−1^ soil, followed by concentrations above 2500 fg g^−1^ soil. The lowest share was indicated for the pathogen DNA below the limit of quantification.

**Figure 2:**
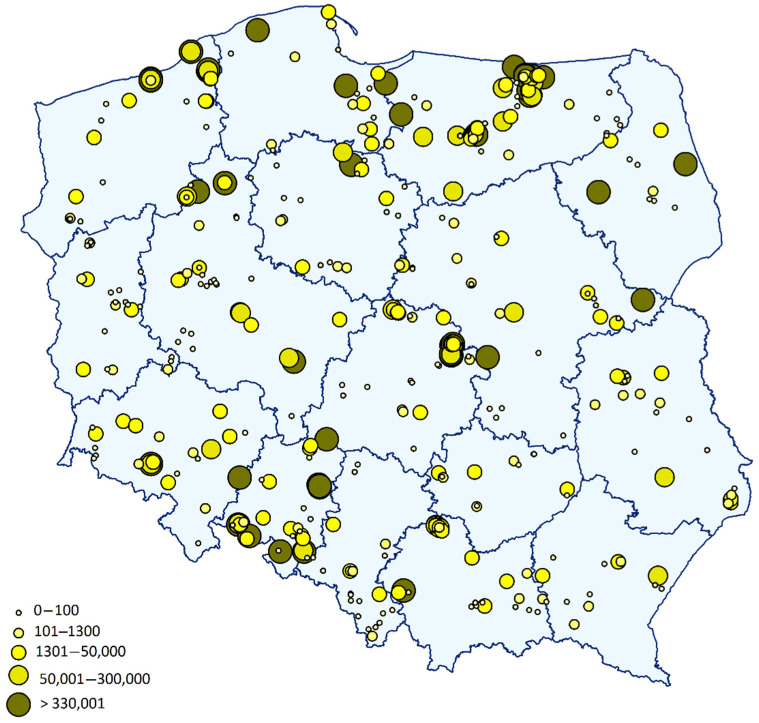
Real-time PCR-based quantification of *Plasmodiophora brassicae* at sampling points across 16 provinces of Poland. Increasing diameter of the circle and its colour correspond to the number of gene copies g^−1^ soil, as indicated in the legend.

DNA of *P. brassicae* was detected in 62% of all tested samples with a maximum pathogen load as high as 1.7 × 10^9^ gene copies g^−1^ soil which is the equivalent of 7.7 × 10^8^ spores. In 38% of the tested fields (165 fields), *P. brassicae* DNA was not detected or the number of gene copies was below the quantification limit. In 14% of the fields (62 fields), the DNA level was lower than 1300 gene copies g^−1^ soil, corresponding to approx. 3000 spores g^−1^ soil. Almost 26% of the samples, collected from 112 fields, were contaminated at a level ranging from 1300 to 50,000 gene copies g^−1^ soil. In 22% of tested farms (93 fields), levels above 50,000 gene copies g^−1^ soil were detected and among them on 12% (53 fields) the highest amounts of *P. brassicae*, exceeding 330,000 gene copies g^−1^ soil, was found. The location of sampling points was divided into five categories: <100; 101–1300; 1301–50,000; 50,001–330,000 and >330,001 gene copies g^−1^ soil. The highest concentrations of spores were found in Pomerania as well as Warmia-Masuria, followed by Opole Province and Lower Silesia (Figure 2).

**Figure 3:**
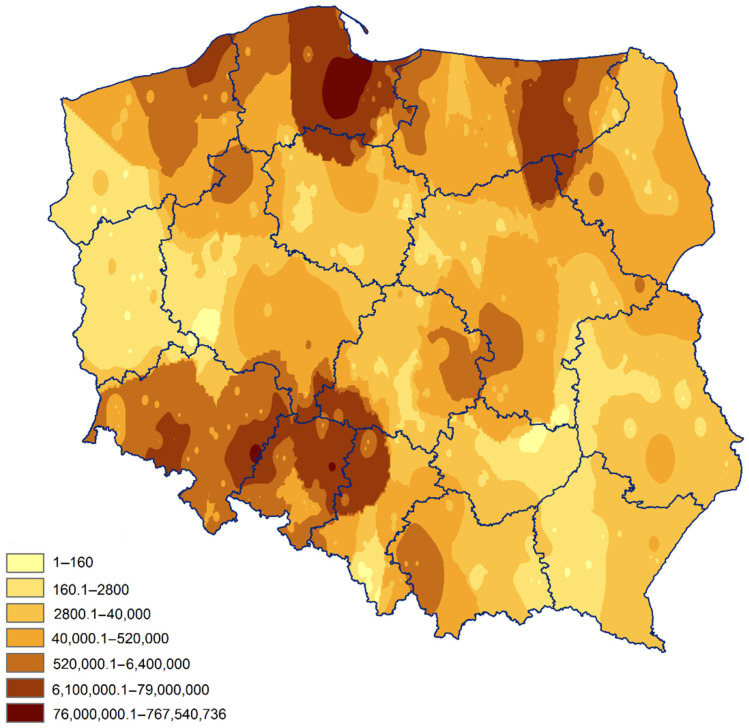
Spore load of *Plasmodiophora brassicae* g^−1^ soil in Poland quantified based on the results of qPCR using ArcGis software and interpolated by Inverse Distance Weight method.

The recalculation of pathogen DNA to the number of spores g^−1^ soil and division into seven arbitrarily chosen, epidemiologically significant categories of spore concentrations (Table S2) revealed that as much as 83% of the samples were associated with a risk of clubroot, and only 17% of the fields could be regarded as potentially safe. Among the ones posing the risk of clubroot, the largest share was obtained for the concentrations between approx. 2800.1–520,000 spores g^−1^ soil. Such spore loads were interpolated for 57.7% area of arable land and they are associated with moderate (2800.1–40,000 spores g^−1^ soil) and high (40,000.1–520,000 spores g^−1^ soil) disease risk. The fields where spore concentrations in soil were >520,000 g^−1^ soil covered as much as 25.3% of the arable area and these areas are correlated with very high disease risk. The highest concentrations of spores were found in Pomerania, Warmia-Masuria, followed by Opole Province and the east part of Lower Silesia (Figure 3). 

**Figure 4:**
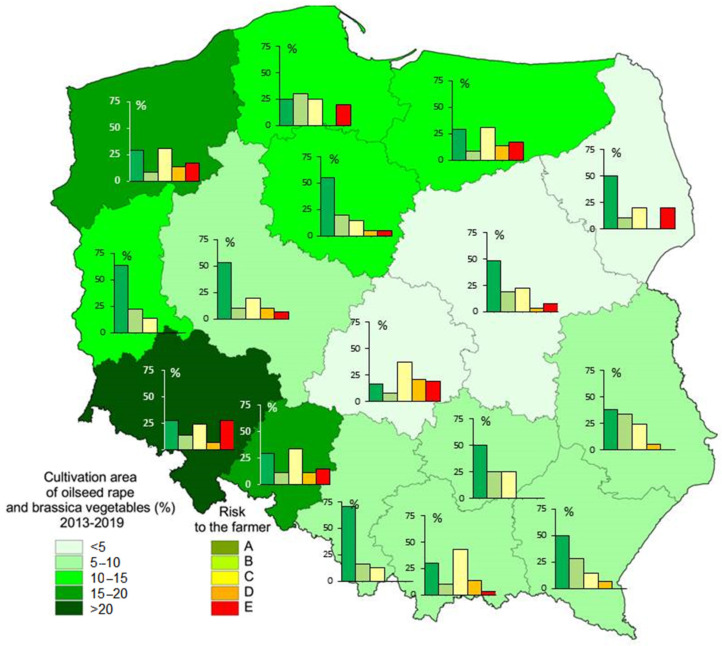
The intensity of oilseed rape and vegetable brassicas cultivation in 16 provinces of Poland and the recommendations for farmers and expected yield losses caused by clubroot if susceptible brassica crops are grown. A: no yield loss caused by clubroot, B: risk of yield loss <10% in susceptible crops, C: risk of crop losses >10% in susceptible crops, resistant cultivars recommended, D: only resistant cultivars are recommended for cultivation, E: cultivation of oilseed rape and vegetable brassicas is not recommended.

From 2013 to 2019, the arable land in Poland averaged 10,648 thousand hectares in total, with varied acreage in different provinces, ranging from 271 thousand hectares in Upper Silesia to 1463 thousand hectares in Greater Poland. Winter and spring oilseed rape as well as Brassicaceae vegetables occupied an area of nearly 906 thousand hectares during these years, winter form of oilseed rape was prevailing. As indicated in Figure 4, they covered the largest percentage of the arable land in Lower Silesia (20%), followed by Opole province (17.8%) and West Pomerania (17.3%). The lowest percentage of brassicas was grown in Lodz Province (4%) and Masovia (4.6%). The highest area of brassica cultivation was in Lower Silesia and Lublin Province whereas the smallest area was in Swietokrzyskie Province, Podlasie, Lesser Poland, Upper Silesia, and Lubusz. Masovia and Lodz Province had the highest production of vegetable brassicas. On average vegetable brassicas occupied 0.33 % of the arable land. The highest proportion of vegetable brassicas was found in Lesser Poland (2.0%) and the lowest in Opole Province (0.05% of the arable land).

Based on the studies carried out by Wallenhammar et al. [51] and Almquist [54] guidelines for farmers have been formulated in respect to the cultivation of vegetable brassicas and oilseed rape in Poland. The guidelines are based on the qPCR detection of *P. brassicae* DNA, measured as the number of gene copies g^−1^ soil. In Sweden, the guidelines have been used commercially by the company Eurofins Agro Testing Sweden AB, Kristianstad, Sweden [55]. For DNA levels below 1300 DNA copies g^−1^ soil (3000 spores g^−1^ soil) the risk of yield loss in susceptible crops is regarded as lower than 10%. At DNA levels ranging from 1300 to 330,000 DNA copies g^−1^ soil (corresponding to 3000 and 750,000 spores g^−1^ soil, respectively) resistant cultivars are recommended. At levels above 330,000 DNA copies g^−1^ soil, the risk of multiplication of soil inoculum is considerable and even the cultivation of clubroot resistant (CR) cultivars of vegetable brassicas and oilseed rape is not recommended. The quantification of *P. brassicae* in Polish soils revealed that CR cultivars should be grown on 53.5% of the area currently under cultivation with Brassicaceae crops.

As indicated in Figure 4, the highest restrictions to the cultivation of oilseed rape and vegetable brassicas due to high incidence of *P. brassicae* DNA in the soil were revealed for Lower Silesia (27.7%) and West Pomerania (23.5%), followed by Pomerania and Podlasie (20% each), Lodz Province (18.5%) as well as Warmia-Masuria (17.3%). In the abovementioned provinces brassica crops are intensively grown, predominantly oilseed rape, but also brassica vegetables (Lodz Province) [56]. The exception from this rule is Podlasie, with its low acreage of oilseed rape and vegetable brassicas (Table S2).

**Figure 5:**
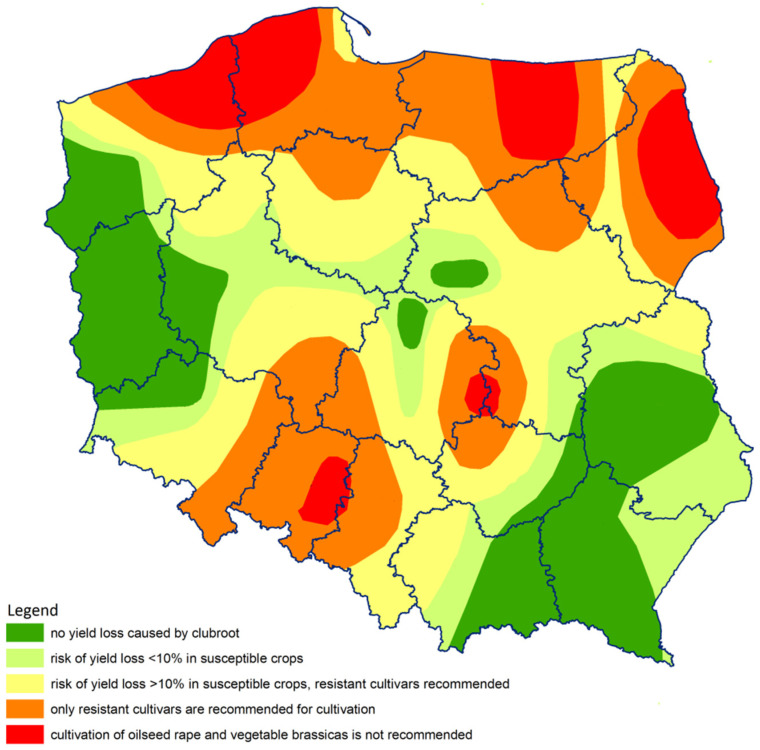
The recommendations for the cultivation of oilseed rape and vegetable brassicas based on the quantification of *Plasmodiophora brassicae* in Polish agricultural soils, according to the guidelines of Wallenhammar et al. [51] and Almquist [54]. Map was prepared using ArcGis software, interpolated by Inverse Distance Weight method.

The map prepared using ArcGis software with the data interpolated by Inverse Distance Weight method indicates that the highest probability of brassica crop failure due to clubroot is in Pomerania, Warmia-Masuria, Podlasie and Opole Province as well as the eastern part of Upper Silesia (Figure 5); all of them, apart from Podlasie, intensively grow oilseed rape. The other regions vulnerable to the pathogen are the brassica vegetable growing areas in Central Poland (Figure 5).

## 3. Discussion

In this study, for the first time, DNA-based screening was performed to monitor the occurrence of *P. brassicae* in agricultural soils used for the cultivation of oilseed rape and vegetable brassicas throughout Poland. Real-time qPCR assays enabled rapid and reliable determination and quantification of the pathogen in farmers’ fields. In the years 2013 to 2019, 431 soil samples were collected from agricultural soils used to grow oilseed rape and Brassicaceae vegetables, across all sixteen provinces of Poland. On average 27 soil samples, varying from 10 (Podlasie) to 58 (Warmia-Masuria), were collected per province. It was found that *P. brassicae* is currently present in every province of Poland. We have detected considerable differences within and between provinces concerning the concentration of *P. brassicae* DNA. In total, as much as 61.9% (267) of the fields in Poland were tested positive for the presence of the pathogen.

It is known that clubroot is found throughout the world wherever Brassica crops are grown [2]. The first report concerning the frequency of clubroot infestation was published in 1981 and covered 6 million hectares of the world [57]. Clubroot disease was then reported in 18 countries with a mean infection rate of 11%. Since then, canola/rapeseed cultivation has expanded significantly. It was confirmed that clubroot is present in Australia and Oceania, Europe, Asia, and the Americas [57,58], but the exact sites affected by the disease are not always reported. Field tests for the presence of *P. brassicae* spores have been carried out in countries including Canada [59], Sweden [60], Germany [16], United Kingdom [61], and the Czech Republic [62]. 

The pathogen can accumulate in a wide variety of soil types. In Canada, a survey for clubroot was initiated at the University of Alberta in 2004 and has been continued until the present. It showed a steady increase in the number of clubroot-infested fields across this province [59]. In 2013, testing of soil samples on over 200 fields in Saskatchewan and Manitoba performed in the absence of clubroot symptoms yielded 3 positive samples (1.5%) [63]. The recently developed ClubrootTracker map (clubroottracker.ca) is freely available and it helps to track *P. brassicae* with the possibility to add new data from the whole world [64]. 

In Sweden, commercially available services for farmers and researchers based on molecular analysis of field samples have been developed within the Biological Soil Mapping (BioSoM) project [60]. The results from 2013 showed that *P. brassicae* DNA was detected in 60% of 45 fields sampled in Scania, south Sweden [60]. In Germany, clubroot has spread to all regions of the country [65]. The detection of 49 new *P. brassicae*-infested fields in 2012–2015 suggests that it is more widespread in German fields than previously known [16]. A survey of oilseed rape fields carried out in Scotland in 2008 and 2009 showed that over half of the tested fields were infected with clubroot. Soil samples were also taken in England and Wales, mainly from farms where clubroot was already found, but a few new cases of pathogen presence were identified [61]. In the Czech Republic, the first occurrence of clubroot on oilseed rape was recorded in 2011 [62]. A serious infestation was found on 44 farms, mainly in the north and northeast of the country; the disease has since spread in the Brassicaceae growing areas [15,62].

In Poland, thus far, Jedryczka et al. [12,13] and Korbas et al. [66] performed studies based on soil bioassay tests in the Greater Poland province, located in the central-west part of the country, as well as on agricultural soils on the Polish–Ukrainian and Polish–Belarussian borders. In the study conducted in Greater Poland, soil samples were obtained from 31 rural and four urban counties (in total, 196 samples). The pathogen was not detected in soils from municipal counties, but it was found in eight rural counties (25.8%). A random sampling of agricultural soils on the Polish-Ukrainian and Polish-Belarussian borders also identified fields infested by *P. brassicae* (6% and 22%, respectively). However, until now, a detailed country-wide assessment of *P. brassicae* spread in agricultural soil samples has not been reported in Poland and the current study fills this gap. 

The severity of the infestation and symptom expression increases with the intensity and frequency of crop production [20]. In Lower Silesia, where the percentage of the Brassicaceae cultivation area was the highest, the intensity of brassica crop cultivation coincided with the greatest concentration of *P. brassicae* in the soil. However, in Podlasie, with a low percentage of vegetable brassicas and oilseed rape (4%), the concentration of the pathogen DNA was also high. A plausible explanation is the soil acidification in this province, dropping below pH 4.5 in 20% of agricultural soils, whereas the average soil pH in this province ranges from 4.6 to 5.0 (www.gios.gov.pl/chemizm_gleb/index.php?mod=wyniki&cz=B). According to Jadczyszyn [67], such low soil pH is one of the main factors limiting agricultural production.

In Sweden [68], high infestation was recorded for pH values from 5.2 to 6.6 and it was the highest in clay soils. In Finland [69], clubroot was found in fields with pH values from 5.0 to 7.6 but severe outbreaks (more than 10% of plants showing symptoms) were in fields with pH values of 6.7 or lower. In contrast, in Canada, a weak negative correlation was found between lower soil pH and higher *P. brassicae* infestation [70]. In the studies done by Ricarova et al. [15], this correlation was relatively weak in Poland and non-existent in the Czech Republic. Therefore, the high level of pathogen infestation in Podlasie may also relate to other factors affecting *P. brassicae* propagation, such as soil type and moisture or common types of crop rotation. Kasinatahan [71] pointed out that soil type and organic matter had a limited impact on clubroot development when other conditions were conducive for *P. brassicae*.

Inoculum density affects clubroot severity and seed yield [6,72]. Murakami et al. [73] pointed out that that disease seldom occurred at an inoculum density below estimated 1 × 10^3^ spores g^−1^. In Japan, the soil infestation level of the fields was usually below 10^6^ spores g^−1^ of soil [74]. Hwang et al. [6] reported that at a soil inoculum density of 10^5^ spores, yield losses were about 80%. Strehlow [73] showed that at similar spore concentrations all plants died due to severe clubroot infection. Numerous factors are influencing the ultimate agricultural performance of crops, including the environment, type of the cultivar, agrotechnological treatments, soil type as well as the pathogen itself [75]. The differences may also be due to the properties of particular isolates. It was hypothesized that those derived from Australia and America may have been exposed to a much more limited host range and their virulence towards *B. napus* hosts may be lower [75].

Recommendations for Polish farmers have been elaborated based on the guidelines of Almquist [54], derived from the previous studies performed by Wallenhammar et al. [51]. Soils were assigned to the classes based on the risk of infection depending on the abundance of the pathogen DNA. In Poland, a risk of yield loss lower than 10% in susceptible crops was revealed for 14% of the fields. In 26% of the fields, *P. brassicae* DNA was found at the level corresponding to a risk of crop losses of more than 10% in susceptible crops; in such fields, the farmers are advised to grow clubroot-tolerant or resistant crops. For 22% of tested farms, a high risk of complete crop failures was predicted if susceptible oilseed rape cultivars would be sown. Finally, on 12% of fields, the concentration above 50,000 gene copies g^−1^ soil was found, indicating the highest risk for the cultivation of brassicas, even for resistant cultivars. Growing resistant Brassicaceae plants on such severely clubroot-infested soils can lead to a high pressure on the pathogen population and as a result, risking the selection of pathotypes capable to break resistance mechanisms. For comparison, in Sweden, pathogen DNA at a level of > 50,000 DNA copies g^−1^ soil was found in only five samples—that is 8% of the fields tested in 2014 [60]. In our previous studies, soil samples were collected from 16 clubroot-infested fields of oilseed rape in Poland and 14 in the Czech Republic, and the highest *P. brassicae* resting spore concentration was detected in a Polish soil sample, collected near Tuczno (West Pomerania) where the number of spores exceeded 8 × 10^7^ spores g^−1^ dry soil. In the Czech Republic, the highest concentration (4 × 10^6^ spores g^−1^ dry soil) was found in a sample from Modlibohov near Liberec, in the north of the country, ca. 20 km from the border with Poland [15].

In this study, based on the example of the BioSoM project performed in Sweden [60], we have monitored the incidence and severity of *P. brassicae* in agricultural soils of Poland and we have pinpointed the regions of the highest disease risk. Indications for the cultivation of oilseed rape and brassica vegetables have been formulated, based on recommendations provided by the Swedish researchers [51,54]. For 165 farmers’ fields, no yield losses were expected despite the growth of non-CR cultivars. The expected yield loss predicted for 62 fields was less than 10%, but for another 112 fields, it was >10% if susceptible brassica crops were grown. For 40 fields, the cultivation of CR-varieties was recommended, whereas for 53 fields a break in Brassicaceae cultivation was suggested, to prevent further multiplication of already high soil infestation. Since 2014, the Central Cultivar Testing Station in Slupia Wielka, Poland has already registered ten cultivars of oilseed rape resistant to clubroot. The nature of this trait is not fully known, however, most probably it is based on single resistance genes, therefore it is prone to breaking under high pressure of the pathogen and pathotype variability occurring in Poland [15]. Our study clearly shows that in Poland, intensive preventive actions should be undertaken to reduce the further spread of *P. brassicae,* as over one-fifth of the fields was highly infested by *P. brassicae*.

Clubroot management relies on preventing the introduction of the disease into fields and when it is already present the focus is on reducing its incidence and severity. Due to the longevity of the pathogen, it is worthwhile to check soil for the presence of clubroot spores. As the pathogen infestation in the field soil persists for a long time, knowledge of *P. brassicae* spores distribution remains useful for several seasons. Our database, covering fields across the whole Poland, allowed us to create interpolated clubroot disease risk maps with associated recommendations for the cultivation of oilseed rape and brassica vegetables. Soil mapping can be also an important tool for predicting infection potential and possible yield losses. Moreover, it may help farmers to identify clubroot-infested areas in a defined region. Elaborated soil maps can be used by researchers, commercial companies, students, and extension services for various purposes, including education, advice and guidelines. The identification of agricultural regions with soils infested by *P. brassicae* highlights the need for the adoption of special hygiene measures, such as the compulsory decontamination of field machinery, in defined areas. The recommendations by Almquist [54] used in this study are currently valid. However, they should not be considered as durable and constant, but prone to changes following the implementation of new cultivation practices, the introduction of novel cultivars and the changes in the pathogen population as well as in the environment. This particular study concerning Poland shows a high risk of clubroot disease in all areas of intensive oilseed rape and vegetable brassica cultivation. There is an urgent need for the recognition of the pathotype distribution and the use of the cultivars resistant to the most damaging ones.

## 4. Material and Methods

### 4.1. Soil Sampling

**Figure 6:**
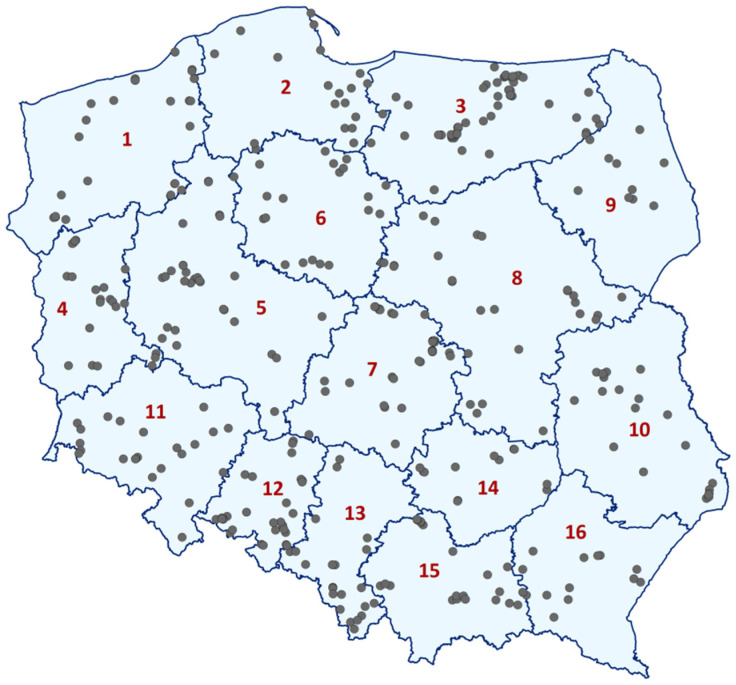
Locations sampled in 2013–2019 in Poland, containing 431 fields in 16 provinces: 1—West Pomerania, 2—Pomerania, 3—Warmia-Masuria, 4—Lubusz, 5—Greater Poland, 6—Kuyavia-Pomerania, 7—Lodz Province, 8—Masovia, 9—Podlasie, 10—Lublin Province, 11—Lower Silesia, 12—Opole Province, 13—Upper Silesia, 14—Swietokrzyskie, 15—Lesser Poland, and 16—Subcarpathia.

Soil samples were collected between 2013 and 2019 from 431 fields across Poland under the cultivation of Brassicaceae crops, *ca*. 30 fields randomly sampled in each region, except for Podlasie (10 fields) and Swietokrzyskie (12 fields), where oilseed rape and vegetable brassicas are less commonly grown. The location of the sampled fields is shown in Figure 6. From each field the sample of approximately 2l, consisting of ca. 40 subsamples (soil cores) was collected along a W-transect. A soil auger with a diameter of 30 mm and a volume of 25 cm^3^ was used to take subsamples representing the top 20 cm of the soil profile. The fields varied in size; in the case of vegetable brassica cultivation they were usually 1 ha or below. Fields of oilseed rape ranged from approximately 10 ha to 50 ha. The number of subsamples was the same (40), regardless of the field size. The location of each tested field was determined using GPS coordinates.

### 4.2. DNA Extraction from Soil

Soil sub-samples were mixed by hand. The plant material and stones were removed. Then, the samples were air-dried at room temperature (20 °C). Then, soil samples were divided into two parts and ground with metal nuts in a paint shaker (2 replicates × 1l), and then sieved through a 20 mm mesh. DNA extraction from soil was done according to the procedure described by Wallenhammar [51]. Briefly, DNA was extracted from 350 mg soil samples (two biological replicates per sample to confirm intersample reproducibility of the assay) using the Fast DNA Spin Kit for Soil (MP Biomedicals, Irvine, CA, USA), and obtained eluate was then further purified using Wizard DNA Clean-Up System (Promega Corporation, Madison, WI, USA) and Illustra MicroSpin S-300 HR Columns (GE Healthcare, Boston, MA, USA). To assess the quality of the isolated DNA, the concentration and purity of the DNA samples were measured on UV/Vis photometer (BioPhotometer plus, Eppendorf, Hamburg, Germany). Only DNA samples with a ratio of the absorption values A260 to A280 between 1.8–2.0 was used. Forty randomly selected DNA samples (ca. 10% of the studied lot) were subjected to electrophoresis on a 1.5% agarose gel and stained with ethidium bromide. All of the samples proved high purity of DNA; therefore, the spectrophotometric evaluation of the samples was considered as a sufficient method of DNA quality confirmation.

### 4.3. Real-Time qPCR

The sequences of primers and probes as well as qPCR conditions, were the same as described by Wallenhammar [51]. The primers PbF 5′-AAA CAA GTC AGC TTG AAT GC-3′ and PbR 5′-TTC GCG CAC AAG CAC TTG-3′ were used to amplify a 103 bp fragment of ribosomal RNA gene (GeneBank accession number AF231027). A probe PbP 5′-CGC GCC ATG CAC TGT TAA ATT G-3′ with VIC as the 5′ terminal reporter dye and TAMRA as 3′ quencher was used to improve qPCR specificity. Real-time quantitative PCR was performed using the 7300 Real-Time PCR System (Applied Biosystems, Waltham, MA, USA) in a total volume of 25 μL. The qPCR reaction mixture included: 1 × TaqMan Universal PCR Master Mix No AmpErase UNG (Life Technologies, Carlsbad, CA, USA), 0.12 μm PbF, 0.6 μm PbR, 0.2 μm PbP and 5 μL DNA template diluted 1:5. The thermal cycling conditions were as followed: pre-PCR denaturation for 10 min at 95 °C, and then 45 cycles consisting of a 15 s denaturation step at 95 °C and 60 s annealing/extension step at 59 °C. All DNA samples were analyzed in duplicate, meaning that four measurements were performed for each soil sample to confirm intra-assay precision. Soil with a known amount of pathogen was used as a positive control in each qPCR run to confirm inter-assay precision and reproducibility. Moreover, a no template (negative) control was included in each qualitative assay. The amount of the pathogen was quantified using a standard curve obtained for a 10-fold dilution series of the plasmid containing the synthetic gene (Eurofins MWG Operon, Ebersberg, Germany) of the *P. brassicae* target sequence.

### 4.4. Quantification of Plasmodiophora Brassicae Spores 

Spores of *P. brassicae* were extracted from Chinese cabbage cv. ´Granaat´ roots infected with five isolates originating from oilseed rape (*Brassica napus* L.) with visible symptoms of clubroot, collected at five regions of Poland (Lower Silesia, Opole Province, Pomerania, Warmia and Masuria, West Pomerania). Roots (5 g) were macerated with MQ water (50 mL) using a blender. The suspension was then filtered through four layers of cheesecloth and diluted 100 times giving final spores concentration between 1.7 × 10^5^ and 5 × 10^5^. Spores were counted under the microscope Optika B-350 (Optika, Ponteranica, Italy) using a Fuchs-Rosenthal hemocytometer (Brand, Wertheim, Germany). DNA from spores was extracted using the same procedure as described previously and quantified using real-time qPCR as described above.

Based on the frequency tabulation for log10 (No_spores) seven categories of spore loads were derived, containing samples with similar values. Each category has been attributed to the probable disease level and yield risk. 

### 4.5. Cultivation Guidelines Based on qPCR Assay

Guidelines for farmers based on the levels of DNA copies g^−1^ soil have been adopted from Wallenhammar et al. [51] and Almquist [54]. For DNA levels lower than 1300 DNA copies g^−1^ soil (3000 spores g^−1^ soil), the risk of yield loss in susceptible crops is regarded as lower than 10%. At DNA levels ranging from 1300 to 330,000 DNA copies g^−1^ soil (corresponding to 3000 and 750,000 spores g^−1^ soil, respectively), resistant cultivars are recommended. At levels above 330,000 DNA copies g^−1^ soil, the risk of multiplication of soil inoculum is considerable hence no cultivation of Brassicaceae crops is advised.

### 4.6. Preparation of Maps 

The maps were generated and interpolation analyses were performed using the ArcGis ver. 10.2 software package (ESRI Polska Sp. z o.o., Warszawa, Poland). Interpolation was performed using the method of Inverse Distance Weight (IDW). The IDW method assumes that the value at any point where there is no measurement is equal to the weighted average value of nearby points. The influence of the measuring points decreases with distance. The greater the weighting factor, the less important the further measuring points are. In this study, the value 2 was applied after previewing the output and examining the cross-validation statistics, as indicated in ArcGIS Desktop Help 9.2 (https://support.esri.com/en/).

### 4.7. Statistical Analysis and Data Integration

Statistical significance of real-time qPCR results was studied using one-way ANOVA (*p* = 0.05) and Duncan’s post hoc test (Statistica 9.0, StatSoft, Warszawa, Poland). The correlation of linear regression comparing microscopic and qPCR quantification of spores was confirmed by Pearson´s correlation coefficient (R = 0.974) at *p* < 0.05. The information about the cultivation area used in the study was obtained from Statistics Poland (https://stat.gov.pl). The results were analyzed for the percentage of oilseed rape and brassica vegetables in relation to the cultivation area in individual provinces. Available statistics were compiled for the years between 2013 and 2019.

## 5. Conclusions

The study revealed that *Plasmodiophora brassicae* is present in agricultural soils of all 16 provinces of Poland. DNA of *P. brassicae* was detected in 62% of the tested fields. The concentration of pathogen DNA measured using real-time qPCR technique reached 1.7 × 10^9^ gene copies g^−1^ soil which is the equivalent of 7.7 × 10^8^ spores. The spore loads in fields cultivated with Brassicaceae crops were mostly high and in 19.5% of the sampled fields, they exceeded 1 × 10^4^ spores g^−1^ soil. The highest clubroot risk occurred in Pomerania, West Pomerania, Warmia-Masuria, east Podlasie, south part of Lower Silesia and north part of Opole Province. The intensity of oilseed rape cultivation in these areas is very high, except for Podlasie, where soils are acidic, which favors the development of *P. brassicae*. Additionally, soils in areas with intensive cultivation of Brassica vegetables located in central Poland were also highly infested by the pathogen. Concluding, the intensification of agricultural and horticultural production of Brassicaceae crops, accompanied by crop rotation shorter than 4 years, promotes the growth and multiplication of *P. brassicae*. The phenomenon observed in Poland is in agreement with previous findings reported from the other regions of Brassicaceae cultivation. Based on the concentration of *P. brassicae* DNA, recommendations for the farmers growing vegetable brassicas and oilseed rape have been formulated, pointing out the areas where CR cultivars are indispensable. The soil sampling followed by real-time qPCR detection of *P. brassicae* can serve as a reliable tool for decision support for the farmers. The method should be implemented as a part of a toolbox used in the integrated management of clubroot worldwide.

## Supplementary Materials

The following are available online at https://www.mdpi.com/2076-0817/9/12/1070/s1, Table S1: Share of arable land in Poland attributed to different concentrations of *Plasmodiophora brassicae* DNA (fg g^−1^ soil) calculated using ArcGIS software, interpolated with Inverse Distance Weight method. Table S2: Share of arable land in Poland attributed to different concentrations of *Plasmodiophora brassicae* resting spores and expected disease risk and yield loss.

## References

[1] Woronin M., Typographia W.F. (1877). Plasmodiophora brassicae—Organism, Pricziniajuszczij Kapustnym Rastienjam Boliezn Izwiestnuju Pod Nazvaniem Kily.

[2] Dixon G.R. (2009). The occurrence and economic impact of *Plasmodiophora brassicae* and clubroot disease. J. Plant. Growth Regul..

[3] Strelkov S.E., Hwang S.F., Manolii V., Turnbull G.D., Fredua-Agyeman R., Hollman K., Kaus S. (2020). Characterization of clubroot (*Plasmodiophora brassicae*) from canola (*Brassica napus*) in the Peace Country of Alberta, Canada. Can. J. Plant. Pathol..

[4] Pageau D., Lajeunesse J., Lafond J. (2006). Impact de l’hernie des crucifères [*Plasmodiophora brassicae*] sur la productivité et la qualité du canola. Can. J. Plant. Pathol..

[5] Strelkov S.E., Manolii V.P., Cao T., Xue S., Hwang S.F. (2007). Pathotype classification of *Plasmodiophora brassicae* and its occurrence in *Brassica napus* in Alberta, Canada. J. Phytopathol..

[6] Hwang S.F., Ahmed H.U., Strelkov S.E., Gossen B.D., Turnbull G.D., Peng G., Howard R.J. (2010). Seedling age and inoculum density affect clubroot severity and seed yield in canola. Can. J. Plant. Sci..

[7] Faggian R., Strelkov S.E. (2009). Detection and measurement of *Plasmodiophora brassicae*. J. Plant. Growth Regul..

[8] Chai A.L., Xie X.W., Shi Y.W., Li B.J. (2014). Research status of clubroot (*Plasmodiophora brassicae*) on cruciferous crops in China. Can. J. Plant. Pathol..

[9] Robak J. (1991). Variability of Plasmodiophora brassicae Wor Pathotypes. Occurring in Poland and Their Pathogenicity to Cultivars and Breeding Lines of Brassica oleracea.

[10] Korbas M., Jajor E., Budka A. (2009). Clubroot (*Plasmodiophora brassicae*)—A threat for oilseed rape. J. Plant. Prot. Res..

[11] Robak J., Gidelska A. (2009). Epidemiology and new possibility of control of *Plasmodiophora brassicae* causal agent of clubroot of cruciferous crop in Poland. Prog. Plant. Prot..

[12] Jedryczka M., Korbas M., Jajor E., Danielewicz J., Kaczmarek J. (2013). Occurrence of *Plasmodiophora brassicae* in Wielkopolska province. Prog. Plant. Prot..

[13] Jedryczka M., Kasprzyk I., Korbas M., Jajor E., Kaczmarek J. (2014). Infestation of Polish agricultural soils by *Plasmodiophora brassicae* along the Polish-Ukrainian border. J. Plant. Prot. Res..

[14] Eckert M., Gout L., Rouxel T., Blaise F., Jedryczka M., Fitt B.D.L., Balesdent M.H. (2005). Identification and characterization of polymorphic minisatellites in the phytopathogenic ascomycete *Leptosphaeria maculans*. Curr. Genet..

[15] Ricarova V., Kaczmarek J., Strelkov S.E., Kazda J., Lueders W., Rysanek P., Manolii V., Jedryczka M. (2016). Pathotypes of *Plasmodiophora brassicae* causing damage to oilseed rape in the Czech Republic and Poland. Eur. J. Plant. Pathol..

[16] Zamani-Noor N. (2017). Variation in pathotypes and virulence of *Plasmodiophora brassicae* populations in Germany. Plant. Pathol..

[17] Strelkov S.E., Hwang S.F., Manolii V.P., Cao T., Fredua-Agyeman R., Harding M.W., Oeng G., Gossen B.D., Mcdonald M.R., Feindel D. (2018). Virulence and pathotype classification of *Plasmodiophora brassicae* populations collected from clubroot resistant canola (*Brassica napus*) in Canada. Can. J. Plant. Pathol..

[18] Konieczny W. (2012). Clubroot is present on 250 thousand hectares. Farmer.

[19] Robak J., Czubatka A., Czajka A. (2014). Integrated pest management of crucifer crops against clubroot. Prog. Plant. Prot..

[20] Dixon G.R. (2009). *Plasmodiophora brassicae* in its environment. J. Plant. Growth Regul..

[21] McDonald M.R., Sharma K., Gossen B.D., Deora A., Feng J., Hwang S.-F. (2014). The role of primary and secondary infection in host response to *Plasmodiophora brassicae*. Phytopathology.

[22] Rashid A., Ahmed H.U., Xiao Q., Hwang S.F., Strelkov S.E. (2013). Effects of root exudates and pH on *Plasmodiophora brassicae* resting spore germination and infection of canola (*Brassica napus* L.) root hairs. Crop. Protect..

[23] Walerowski P., Gündel A., Yahaya N., Truman W., Sobczak M., Olszak M., Rolfe S., Borisjuk L., Malinowski R. (2018). Clubroot disease stimulates early steps of phloem differentiation and recruits SWEET sucrose transporters within developing galls. Plant. Cell.

[24] Malinowski R., Truman W., Blicharz S. (2019). Genius architect or clever thief—How *Plasmodiophora brassicae* reprograms host development to establish a pathogen oriented physiological sink. MPMI.

[25] Nowicki B. (1984). Zróżnicowanie Biologiczne Plasmodiophora brassicae Wor. w Polsce oraz Podatność Uprawianych Roślin Krzyżowych na Wykryte Patotypy Grzyba.

[26] Dixon G.R. (2002). Interactions of soil nutrient environment, pathogenesis and host resistance. Plant. Prot. Sci..

[27] Sharma K., Gossen B.D., McDonald M.R. (2011). Effect of temperature on primary infection by *Plasmodiophora brassicae* and initiation of clubroot symptoms. Plant. Pathol..

[28] Rimmer S.R., Shattuck V.I., Buchwaldt L. (2008). Compendium of Brassica Diseases.

[29] Gossen B.D., Deora A., Peng G., Hwang S., McDonald M.R. (2014). Effect of environmental parameters on clubroot development and the risk of pathogen spread. Can. J. Plant. Pathol..

[30] Rennie D.C., Manoli V.P., Cao T., Hwang S.F., Howard R.J., Strelkov S.E. (2011). Direct evidence of surface infestation of seeds and tubers by *Plasmodiophora brassicae* and quantification of spore loads. Plant. Pathol..

[31] Chai A.L., Li J.P., Xie X.W., Shi Y.X., Li B.J. (2016). Dissemination of *Plasmodiophora brassicae* in livestock manure detected by qPCR. Plant. Pathol..

[32] Niemann J., Kaczmarek J., Książczyk T., Wojciechowski A., Jedryczka M. (2017). Chinese cabbage (*Brassica rapa* ssp. *pekinensis*)—A valuable source of resistance to clubroot (*Plasmodiophora brassicae*). Eur. J. Plant. Pathol..

[33] Donald E.C., Porter I.J. (2009). Integrated control of clubroot. J. Plant. Growth Regul..

[34] Hwang S.F., Howard R.J., Strelkov S.E., Gossen B.D., Peng G. (2014). Management of clubroot (*Plasmodiophora brassicae*) on canola (*Brassica napus*) in western Canada. Can. J. Plant. Pathol..

[35] Dixon G.R. (2014). Clubroot (*Plasmodiophora brassicae* Woronin)—An agricultural and biological challenge worldwide. Can. J. Plant. Pathol..

[36] Fedotova T. (1933). Contribution to the evolution of a method for the evaluation of soil infection with clubroot (*Plasmodiophora brassicae* Wor.). Trudjj Zashchite Rastenii.

[37] Naiki T., Takahashi K., Kageyama K. (1984). The relationship between toot hair infection with *Plasmodiophora brassicae* Wor. and subsequent club formation among cruciferous species. Ann. Phytopathol. Soc. Jpn..

[38] Takahashi K., Yamaguchi T. (1987). Assessment of pathogenicity of resting spores of *Plasmodiophora brassicae* in soil by fluorescence microscopy. Ann. Phytopathol. Soc. Jpn..

[39] Colhoun J. (1957). A technique for examining soil for the presence of *Plasmodiophora brassicae* Woron. Ann. Appl. Biol..

[40] Melville I.E., Hawken R.W. (1967). Soil testing for club root in Devon and Cornwall. Plant. Pathol..

[41] Friberg H. (2015). Persistence of *Plasmodiophora brassicae*. Influence of Non-Host Plants, Soil Fauna and Organic Material. Ph.D. Thesis.

[42] Lange L., Heide M., Hobolth L., Olson L.W. (1989). Serological detection of *Plasmodiophora brassicae* by Dot immunobinding and visualization of the serological reaction by scanning electron microscopy. Phytopathology.

[43] White J.G., Wakeham A.J. (1996). Serological detection of resting spores of *Plasmodiophora brassicae* in soil. EPPO Bulletin..

[44] Wakeham A., Faggian R., Kennedy R. (2008). Development and validation of ‘‘in field’’ detection kits for the clubroot pathogen *Plasmodiophora brassicae*. Plant Pathol. J..

[45] Ito S., Maehara T., Maruno E., Tanaka S., Kameya-Iwaki M., Kishi F. (2008). Development of a PCR-based assay for the detection of *Plasmodiophora brassicae* in soil. J. Phytopathol..

[46] Faggian R., Bulman S.R., Lawrie A.C., Porter I.J. (1999). Specific polymerase chain reaction primers for the detection of *Plasmodiophora brassicae* in soil and water. Phytopathology.

[47] Staniaszek M., Robak J., Marczewski W. (2001). Detection of *Plasmodiophora brassicae* Wor. by bioassay and nested PCR methods. Veg. Crop. Res. Bull..

[48] Wallenhammar A.C., Arwidsson O. (2001). Detection of *Plasmodiophora brassicae* by PCR in naturally infested soils. Eur. J. Plant Pathol..

[49] Cao T., Tewari J., Strelkov S.E. (2007). Molecular detection of *Plasmodiophora brassicae*, causal agent of clubroot of crucifers, in plant and soil. Plant. Dis..

[50] Sundelin T., Christensen C.B., Larsen J., Moller K., Bodker L., Jensen B. (2010). In-planta quantification of *Plasmodiophora brassicae* using signature fatty acids and real time PCR. Plant Dis..

[51] Wallenhammar A.C., Almquist C., Jonsson A. (2012). In-field distribution of *Plasmodiophora brassicae* measured using quantitative real-time PCR. Plant Pathol..

[52] Li J.P., Li Y., Shi Y.X., Xie X.W., A-li C., Li B.J. (2013). Development of a Real-Time PCR assay for *Plasmodiophora brassicae* and its detection in soil samples. J. Integr. Agric..

[53] Kaczmarek J., Irzykowski W., Burzyński A., Jędryczka M. (2014). The detection of *Plasmidiophora brassicae* using Loop-mediated isotermal DNA amplification. Acta Agtobot..

[54] Almquist C. (2016). Monitoring Important Soil-Borne Plant Pathogens in Swedish Crop Production Using Real-Time PCR. Ph.D. Thesis.

[55] Eurofins Agro Testing Sweden AB (2016). Klumprotsjuka. Analys av *Plasmodiphora brassicae* i Jord med Snabb och Specific Kvantifiering med DNA-Baserad Teknik. https://cdnmedia.eurofins.com/european-east/media/681411/folder-rapssjukdomar20150707.pdf.

[56] (2019). National Agricultural Census 2019—Horticultural Crops.

[57] Crête R. (1981). Worldwide importance of clubroot. Clubroot Newsl..

[58] Botero A., García C., Gossen B.D., Strelkov S.E., Todd C.D., Bonham-Smith P.C., Pérez-López E. (2019). Clubroot disease in Latin America: Distribution and management strategies. Plant. Pathol..

[59] Gossen B.D., Strelkov S.E., Manolii V.P., Rennie D.C., Cao T., Hwang S.F., Peng G., McDonald M.R. (2015). Spread of *Plasmodiophora brassicae* on canola in Canada, 2003–2014: Old pathogen, new home. Can. J. Plant. Pathol..

[60] Wallenhammar A.C., Gunnarson A., Hansson F., Jonsson A. (2016). Quantification of *Plasmodiophora brassicae* using a DNA-based soil test facilitates sustainable oilseed rape production. Plants.

[61] McGrann G.R.D., Gladders P., Smith J.A., Burnett F. (2016). Control of clubroot (*Plasmodiophora brassicae*) in oilseed rape using varietal resistance and soil amendments. Field Crops Res..

[62] Kazda J., Ricarova V., Prokinova E., Grimova L., Baranyk P. (2013). Nádorovitost kořenů brukvovitých ohrožuje ozimou řepku. Uroda.

[63] Strelkov S.E., Hwang S.F. (2014). Clubroot in the Canadian canola crop: 10 years into the outbreak. Can. J. Plant. Pathol..

[64] Muirhead K., Todd C.D., Wei Y., Bonham-Smith P., Pérez-López E. (2020). ClubrootTracker: A Resource to Plan a Clubroot-Free Farm. Plant. Health Prog..

[65] Diederichsen E., Frauen M., Ludwig-Müller J. (2014). Clubroot disease management challenges from a German perspective. Can. J. Plant. Pathol..

[66] Korbas M., Jajor E., Kaczmarek J., Perek A., Jedryczka M. (2014). Infestation of Polish agricultural soils by *Plasmodiophora brassicae* on the Polish-Belarussian border in Podlasie province. IOBC/Wprs Bulletin..

[67] Jadczyszyn J. (2013). Ocena użytkowania gruntów na obszarach specyficznych oraz charakterystyka czynników ograniczających produkcję rolniczą. Rolnictwo na Obszarach Specyficznych.

[68] Wallenhammar A.C. (1996). Prevalence of *Plasmodiophora brassicae* in a spring oilseed rape growing area in central Sweden and factors influencing soil infestation levels. Plant. Pathol..

[69] Rastas M., Latvala S., Hannukkala A. (2012). Occurrence of *Plasmodiophora brassicae* in Finnish turnip rape and oilseed rape fields. Agric. Food Sci..

[70] Gossen B.D., Kasinathan H., Cao T., Manolii V.P., Strelkov S.E., Hwang S.F., McDonald M.R. (2013). Interaction of pH and temperature affect infection and symptom development of *Plasmodiophora brassicae* in canola. Can. J. Plant. Pathol..

[71] Kasinathan H. (2012). Influence of pH, Temperature, and Biofungicides on Clubroot of Canola. Ph.D. Thesis.

[72] Strehlow B., de Mol F., Struck C. (2015). Risk potential of clubroot disease on winter oilseed rape. Plant. Dis..

[73] Murakami H., Tsushima S., Shishido Y. (2000). Soil suppressiveness to clubroot disease of Chinese cabbage caused by *Plasmodiophora brassicae*. Soil Biol. Biochem..

[74] Tsushima S., Murakami H., Akimoto T., Katahira M., Kuroyanagi Y., Shishido Y. (2010). A practical estimating method of the dose-response curve between inoculum density of *Plasmodiophora brassicae* and the disease severity for long-term IPM strategies. JARQ.

[75] Donald E., Cross S., Lawrence J., Porter I. (2006). Pathotypes of *Plasmodiophora brassicae*, the cause of clubroot, in Australia. Ann. Appl. Biol..

