# Supplementary material for: Country-Wide qPCR Based Assessment of Plasmodiophora brassicae Spread in Agricultural Soils and Recommendations for the Cultivation of Brassicaceae Crops in Poland
Source: Pathogens. 2020 Dec 20;9(12):1070. doi: 10.3390/pathogens9121070 (PMC7766057; doi:10.3390/pathogens9121070)
Supplement: Supplementary file 1 [file pathogens-09-01070-s001.pdf]

Supplementary Materials

Table S1. Share of arable land in Poland attributed to different concentrations of *Plasmodiophora brassicae* DNA (fg g^{-1} soil) calculated using ArcGIS software, interpolated with Inverse Distance Weight method.

No.	DNA of *P. brassicae* (fg per g^{-1} soil)	Area [ha]	% area
1	1-4.9	631,805	1.9
2	5.0 - 24.9	1,767,172	5.7
3	25.0 - 74.9	1,409,909	4.5
4	75.0 - 249.9	3,463,130	11.1
5	250.0 - 999.9	6,516,259	20.9
6	1000.0 - 2499.9	5,068,537	16.2
7	2500.0 - 9999.9	6,310,807	20.2
8	10,000.0 - 2,859,171	6,079,889	19.5

Table S2. Share of arable land in Poland attributed to different concentrations of *Plasmodiophora brassicae* resting spores and expected disease risk and yield loss.

No.	No of spores g^{-1} soil	% area	Clubroot risk
1	1-160	0.9	None
2	160.1-2,800	16.1	Low
3	2,800.1-40,000	28.2	Moderate
4	40,000.1-520,000	29.5	High
5	520,000.1-6,400,000	15.0	Very high
6	6,400,000.1-76,000,000	9.5	Extreme
7	76,000,000.1-767,540,736	0.8	Unavoidable
